# Identification and Function of Apicoplast Glutaredoxins in *Neospora caninum*

**DOI:** 10.3390/ijms222111946

**Published:** 2021-11-04

**Authors:** Xingju Song, Xu Yang, Zhu Ying, Heng Zhang, Jing Liu, Qun Liu

**Affiliations:** 1 National Animal Protozoa Laboratory, College of Veterinary Medicine, China Agricultural University, Beijing 100083, China; 18728153735@163.com (X.S.); bs20193050500@cau.edu.cn (X.Y.); YZyingzhu@163.com (Z.Y.); zhrealm@163.com (H.Z.); liujingvet@cau.edu.cn (J.L.); 2 Key Laboratory of Animal Epidemiology of the Ministry of Agriculture, College of Veterinary Medicine, China Agricultural University, Beijing 100083, China

**Keywords:** *Neospora caninum*, apicoplast, glutaredoxin S14, glutaredoxin C5

## Abstract

Glutaredoxins (GRXs), important components of the intracellular thiol redox system, are involved in multiple cellular processes. In a previous study, we identified five GRXs in the apicomplexan parasite, *Neospora caninum*. In the present study, we confirmed that the GRXs S14 and C5 are located in the apicoplast, which suggests unique functions for these proteins. Although single-gene deficiency did not affect the growth of parasites, a double knockout (Δ*grx* S14Δ*grx* C5) significantly reduced their reproductive capacity. However, there were no significant changes in redox indices (GSH/GSSG ratio, reactive oxygen species and hydroxyl radical levels) in double-knockout parasites, indicating that *grx* S14 and *grx* C5 are not essential for maintaining the redox balance in parasite cells. Key amino acid mutations confirmed that the Cys^203^ of *grx* S14 and Cys^253/256^ of *grx* C5 are important for parasite growth. Based on comparative proteomics, 79 proteins were significantly downregulated in double-knockout parasites, including proteins mainly involved in the electron transport chain, the tricarboxylic acid cycle and protein translation. Collectively, GRX S14 and GRX C5 coordinate the growth of parasites. However, considering their special localization, the unique functions of GRX S14 and GRX C5 need to be further studied.

## 1. Introduction

*Neospora caninum* is an obligate, intracellular, apicomplexan parasite that is found worldwide. This parasite causes spontaneous abortion in cattle and neural system dysfunction in dogs and results in major economic losses in the breeding industry [1,2,3]. During its life cycle, *N. caninum* is exposed to various oxidative stresses, and the parasites develop complex redox networks to maintain redox balance in different stages [4]. Glutaredoxins (GRXs) are ubiquitous oxidoreductases that maintain a cellular redox balance with the thioredoxin family and catalyse thiol-disulphide exchange reactions by utilizing glutathione (GSH) [5]. The number and localization of GRXs differ by species. Humans have four GRXs, which are located in the cytoplasm, nucleus and mitochondria; yeast possesses seven GRXs, located in the cytoplasm, nucleus, mitochondria and endoplasmic reticulum/Golgi [6]. Structurally, GRXs are composed of four β-sheets and three α-helices, with the β-sheets surrounded by α-helices [6]. GRXs are divided into monothiol (CXXS) GRXs and dithiol (CXXC) GRXs depending on the number of cysteine residues [7,8]. GRXs are involved in DNA/RNA synthesis, Fe–S cluster assembly, cell signal transduction, apoptosis and cell proliferation [5,6].

In parasites, GRXs are distributed in different subcellular compartments. For example, *Trypanosoma brucei* has five GRXs: two dithiol TbGRXs (TbGRX1 and TbGRX2) and three monothiol GRXs. GRX1 contains the same CPYC active site as human GRX1 but exhibits a greater amino acid identity (39%) with the human mitochondrial GRX2 (CSYC active site). TbGRX1 coordinates iron–sulphur clusters [9]. TbGrx2 is not essential in vitro or in vivo during the bloodstream stage, but, under fever-like conditions in a mammalian host, TbGrx2 deficiency leads to an increase in thermotolerance. In the procyclical stage, TbGrx2 deficiency significantly affects the morphology of the parasite and leads to irreversible proliferative arrest [10]. The three groups of monothiol GRXs localize to the mitochondria and cytoplasm and are related to the synthesis of iron–sulphur clusters [11]. Additionally, *Trypanosoma cruzi* GRX (TcGRX) is linked to apoptosis-like cell death during infection. In the amastigote stage, the overexpression of TcGRX increases its general resistance to oxidative damage and intracellular replication [12]. *Plasmodium falciparum* expresses three monothiol GRX-like proteins (GLP1, GLP2, GLP3), which localize to the cytoplasm and mitochondria. *P. falciparum* also has one typical dithiol GRX (PfGRX1), which localizes to the cytoplasm [13]. To further elucidate the redox-based, parasite–host cell interactions and the mechanisms of antimalarial action, the redox-sensitive, green, fluorescent protein is coupled to human Grx 1 (hGrx1-roGFP2), with pH and glutathione-dependent redox potential in different subcellular compartments detected via the targeted transfer of hGrx1-roGFP2 into the parasite cytoplasm, mitochondria, or apicoplast [4,14,15].

Our previous study found that *N. caninum* has five GRXs (GRX1, GRX3, GRX S14, GRX C5 and GRX5). GRX1 and GRX3 are located in cytoplasm, and GRX1 deficiency resulted in a marked reduction in the secretion of microneme proteins, significantly affecting the invasion and egress processing of the parasites [16]. In contrast, GRX5 localized to the mitochondria (unpublished), whereas GRX S14 and GRX C5 have yet to be explored. In the present study, we (i) identified two GRXs (GRX S14 and GRX C5) in *N. caninum* located in the apicoplast, (ii) verified that single-gene deletion does not affect the growth of parasites but that double-gene deletion slows their growth, (iii) confirmed that the cysteines of the CXXC/CXXS motif in GRX S14 and GRX C5 are important for parasite growth, and (iv) found that the levels of the electron transport chain (ETC) and tricarboxylic acid cycle (TCA) cycle proteins in Δ*grx* S14 Δ*grx* C5 parasites were downregulated.

## 2. Results

**Figure 1:**
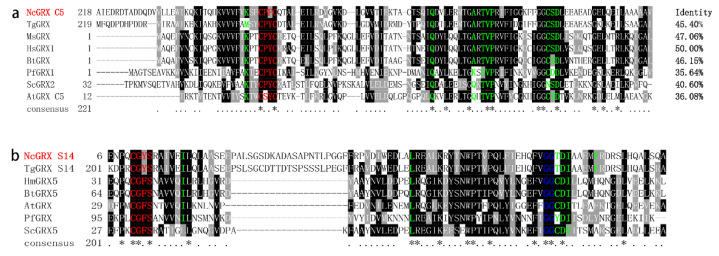
Sequence–structural alignment of the GRX C5 and GRX S14 proteins. (**a**) Alignment of the deduced amino acid sequence of GRX C5 with homologues from other species. The percent homology of GRX C5 with each glutaredoxin is shown at the end of the alignment. Regions of high identity and similarity between glutaredoxin sequences are shown as black and grey columns, respectively. Active site residues are marked with a red letter, and residues involved in interactions with GSH are marked with a green letter. (**b**) Alignment of the deduced amino acid sequence of GRX S14 with homologues from other species. The “*” represent highly conserved amino acid residues.

In this study, we found that GRX C5 has a typical CXXC motif and is a dithiol GRX; GRX S14 has a typical CXXS motif and is a monothiol GRX (Figure 1a). Sequence alignment shows that GRX C5 and GRX S14 has the highest homology with *Toxoplasma gondii* GRX C5 and GRX S14, respectively (Figure 1a,b). According to the tertiary structure prediction, GRX C5 contains a GSH binding motif (CPYC, TVP, CSD motif, Lys and Gln/Arg residues) (Figure S1a), and GRX S14 possesses the GSH binding motif of CGYS, as well as Ile19, Leu60 and Arg94 residues (Figure S1b).

### 2.1. GRX S14 and GRX C5 Localize to the Apicoplast

**Figure 2:**
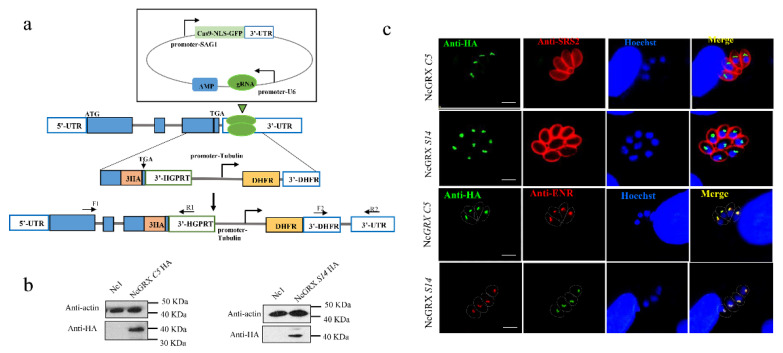
Cellular localization of GRX S14 and GRX C5. (**a**) Strategy for constructing GRX S14-HA and GRX C5-HA parasites. (**b**) Western blotting indicated that the HA tag was successfully added. αHA was used to detect GRX S14 and GRX C5; mouse anti-actin was used as a control. (**c**) IFA indicated GRX S14 and GRX C5 to both be distributed in the apicoplasts of parasites. αHA was used to detect GRX S14 and GRX C5 (green), whereas rabbit anti-SRS2 (red) served as a parasite surface marker. Rabbit anti-ENR (red) was used as an apicoplast marker, and the nuclear DNA was stained with Hoechst (blue) (bar = 5 μm).

To investigate the localization of GRX S14 and GRX C5, we introduced a haemagglutinin (HA) epitope tag at the C-terminus of GRX S14 and GRX C5 in the *N. caninum* wild-type (WT) strain (Nc1) (Figure 2a). Western blotting verified the expected molecular masses of ~40 kDa for GRX S14-HA and GRX C5-HA (Figure 2b). An immunofluorescence assay (IFA) revealed that GRX S14 and GRX C5 localized to the apicoplast (Figure 2c).

### 2.2. GRX S14 and GRX C5 Together Affect the Growth of Parasites, and Their Function Depends on the CXXC/CXXS Motif

**Figure 3:**
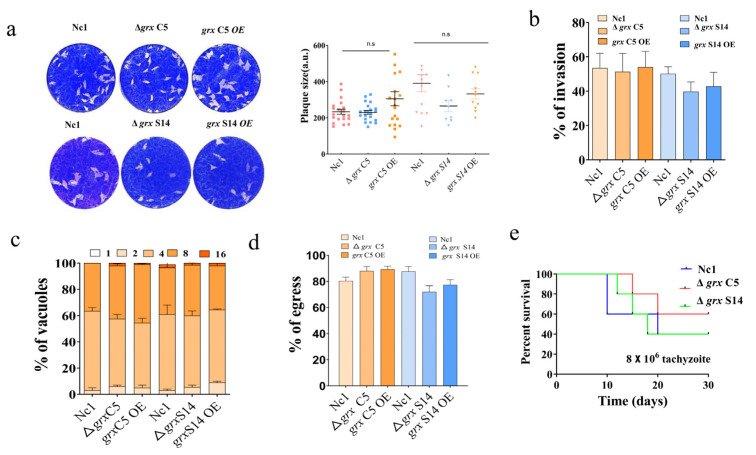
Lack of GRX S14 and GRX C5 alone did not affect the growth of parasites. (**a**) Plaque assays comparing growth of wild-type, knockout and overexpression parasites. Each well contained 300 parasites, and plaques were stained for 9 days. Plaque areas were counted by randomly selecting at least 20 plaques and measuring the pixel point with Photoshop C6S software (Adobe, San Jose, CA, USA). Data were compiled from three independent experiments. (**b**) A total of 1 × 10^5^ parasites were inoculated on human foreskin fibroblast (HFF) cells in 12-well plates and cultured for 24 h. IFA was performed with anti-NcSRS2 antibodies and Hoechst staining. The invasion ratio of wild-type, knockout, overexpression and complementary parasites was based on the number of parasite-infecting cells divided by the number of total cells in one horizon. Data are the mean ± SD (error bars) of three independent experiments. Statistical analysis showed no significant difference (Δ*grx* C5, *F*_(2, 6)_ = 0.06720, *p* = 0.9357; Δ*grx* S14, *F*_(2, 6)_ = 2.238, *p* = 0.1879). (**c**) Intracellular replication of different parasite strains was compiled from three separate assays, with 100 total PVs of each strain counted in each assay. Statistical analysis showed no change (Δ*grx* C5, *F*_(8, 15)_ = 0.6462, *p* = 0.7287; Δ*grx* S14, *F*_(8, 15)_ = 0.6732, *p* =0.7080). (**d**) The egress ability of parasites was assessed after treatment with the calcium ionophore A23187. IFA was employed to detect the integrity of the parasitophorous vacuole (PV). The average number of ruptured PVs was determined by counting 100 random vacuoles per slide. Statistical analysis showed no significant difference (Δ*grx* C5, *F*_(2, 6)_ = 2.734, *p* = 0.1432; Δ*grx* S14, *F*_(2, 6)_ = 3.622, *p* = 0.0930). (**e**) Mouse survival after infection with different strains. BALB/c mice (*n* = 5) were injected intraperitoneally with 8 × 10^6^ doses of parasites. Statistical analysis was performed using the survival curve of GraphPad Prism (San Diego, CA, USA).

To investigate the function of the GRX S14 and GRX C5 proteins, we generated two single-gene knockout strains (Δ*grx* C5 and Δ*grx* S14) and overexpression strains through CRISPR/Cas9-mediated homologous recombination (Supplementary Figure S1c,d). All strains were validated using PCR. The plaque formation ability of these strains was analysed, and the plaque assays showed no obvious difference among the Nc1, single-gene knockout and overexpression strains (Figure 3a) (Δ*grx* C5, *F*_(2, 56)_ = 3.104, *p* = 0.0527; Δ*grx* S14, *F*_(2, 30)_ = 2.500, *p* = 0.0990). Moreover, the steps of the lytic cycle (invasion, intracellular replication and egress) were not significantly affected in the gene-edited strains (Figure 3b–d).

**Figure 4:**
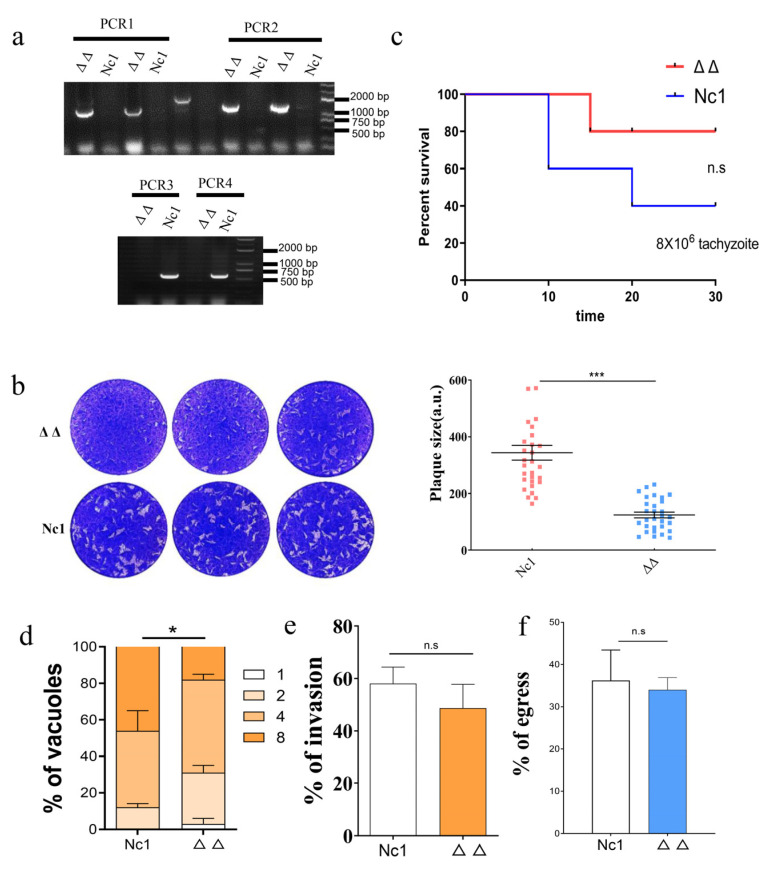
Lack of GRX S14 and GRX C5 together affected the growth of parasites. (**a**) PCR identification of the Δ*grx* S14 Δ*grx* C5 strain. PCR1 and PCR2 suggest successful homologous integration; in PCR3 and PCR4, fragments of *grx* S14 and *grx* C5 were amplified. (**b**) Plaque assay comparing the growth of wild-type and knockout parasites. (**c**) Mouse survival after infection with different strains. (**d**) Intracellular replication of Δ*grx* S14 Δ*grx* C5 compared with Nc1 parasites. Asterisks indicate statistically significant results. (**e**) Invasion assay of Δ*grx* S14 Δ*grx* C5 and Nc1 parasites. (**f**) The egress ability of Δ*grx* S14 Δ*grx* C5 and Nc1 parasites. Statistical analysis was performed using GraphPad Prism (San Diego, CA, USA). n.s: not significant means, *** *p* < 0.001, and * *p* < 0.01.

Because the single-gene knockout strains of *N. caninum* did not exhibit altered growth, we hypothesized that the GRX S14 and GRX C5 proteins had a synergistic effect on the growth of the parasites. To test this hypothesis, we constructed double-gene knockout strains (Δ*grx* C5Δ*grx* S14). PCR verified the successful construction of the Δ*grx* C5Δ*grx* S14 strain (Figure 4a), and a significant reduction in plaque formation size was observed in the Δ*grx* C5Δ*grx* S14 parasites compared with the Nc1 parasites (Figure 4b) (*t*-test: t_(58)_ =  7.758, *p* <  0.0001). We further evaluated the influence on Δ*grx* C5Δ*grx* S14 parasite growth in vivo. The survival rate of mice infected with the Δ*grx* C5Δ*grx* S14 parasites was 80%, and that of mice infected with Nc1 was 40%, indicating a significantly reduced pathogenicity for the Δ*grx* C5Δ*grx* S14 parasites in mice (Figure 4c). Subsequently, we compared the steps of the lytic cycle (invasion, intracellular replication and egress) between the Nc1 and Δ*grx* C5Δ*grx* S14 parasites. Although the intracellular replication of the Δ*grx* C5Δ*grx* S14 parasites was significantly reduced (Figure 4d) (*F*_(3, 8)_ = 5.005, *p* = 0.0305), the invasion and egress were not affected (Figure 4e,f) (*t*-test: t_(2)_ = 1.901, *p* = 0.1977; t_(2)_ = 0.5156, *p* = 0.6575).

**Figure 5:**
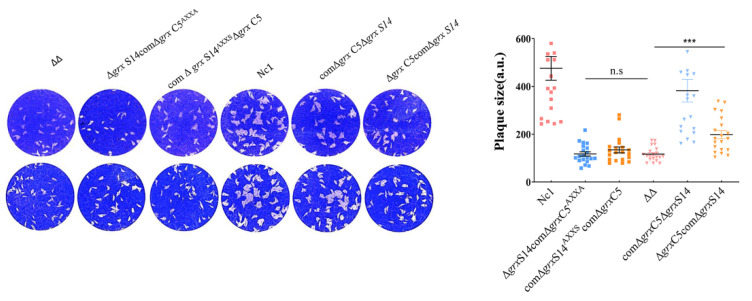
Phenotypes of strains with mutations of key amino acids. Plaque assay comparing growth of the comΔ*grx* S14^AXXS^Δ*grx C5,* Δ*grx* S14comΔ*grx* C5^AXXA^, comΔ*grx* S14Δ*grx* C5, Δ*grx* S14comΔ*grx* C5, Δ*grx* S14Δ*grx* C5 and Nc1 parasites. n.s: not significant means, *** *p* < 0.001.

To assess the importance of the putative CXXS active site of GRX S14 and the CPFC active site of GRX C5, we used ΔΔ*grx S14 grx C5* as the base strain and generated a complementary *grx* S14 sequence with a mutation of the cysteine in the CXXS active site to alanine at the UPRT site (comΔ*grx* S14^AXXS^Δ*grx* C5). In the same way, we constructed the *grx* C5 mutant strain (Δ*grx* S14comΔ*grx* C5^AXXA^), *grx* C5 complemented strain (Δ*grx* S14comΔ*grx* C5) and *grx* S14 complemented strain (comΔ*grx* S14Δ*grx* C5). The phenotype assays showed that complementing the full sequence of *grx* S14 or *grx* C5 could restore the growth ability of the parasites (Figure 5) (*F*_(2, 57)_ = 21.77, *p* < 0.0001), but complementing the mutant sequence could not restore growth (*F*_(2, 57)_ = 1.126, *p* = 0.3316).

### 2.3. Redox Homeostasis of Δgrx C5Δgrx S14 Parasites Was Not Affected

**Figure 6:**
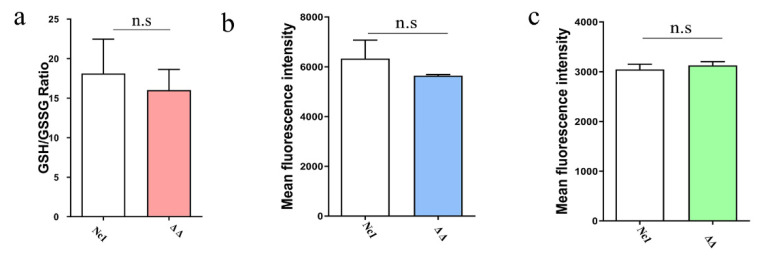
*grx* C5 and *grx* S14 double-gene deficiency did not affect the levels of GSH, ROS and OH. (**a**) Δ*grx* S14Δ*grx* C5 and Nc1 parasites were collected and lysed by three cycles of freezing in liquid nitrogen and thawing at 37 °C. The supernatant of each sample was collected for GSH and GSSG measurement. The GSH/GSSG ratio was calculated, as represented by bar charts according to three independent experiments. (**b**) Reactive oxygen species (ROS) levels of parasites under oxidative stress were determined by FACS analysis using DCFH-DA, whereby the mean fluorescence intensity reflected the ROS level in parasites. (**c**) The hydroxyl radical (OH) content was detected by FACS analysis, with the mean fluorescence intensity reflecting the hydroxyl radical level. n.s: not significant means.

As GRXs play crucial roles in redox-dependent signalling pathways by utilizing GSH as a direct electron donor [6], we measured the GSH/GSSG content in Δ*grx* C5Δ*grx* S14 parasites. The results showed no significant differences in the GSH/GSSG ratio between Δ*grx* C5Δ*grx* S14 parasites and Nc1 parasites (Figure 6a) (*t*-test: t_(2)_ = 0.3968, *p* = 0.7299).

GRXs play an important role in the reactive oxygen species (ROS) antioxidant system. To examine redox homeostasis, we compared the ROS and hydroxyl radical (OH) levels between Δ*grx* C5Δ*grx* S14 and Nc1 parasites and found no significant differences between the two strains (Figure 6b,c) (*t*-test: t_(2)_ = 0.9853, *p* = 0.4284; t_(2)_ = 2.371, *p* = 0.1411). These data indicate that the double-gene deficiency of GRX C5 and GRX S14 does not affect the redox homeostasis of tachyzoites.

### 2.4. GRX S14 and GRX C5 Are not Involved in the Control of Protein Trafficking to the Apicoplast

**Figure 7:**
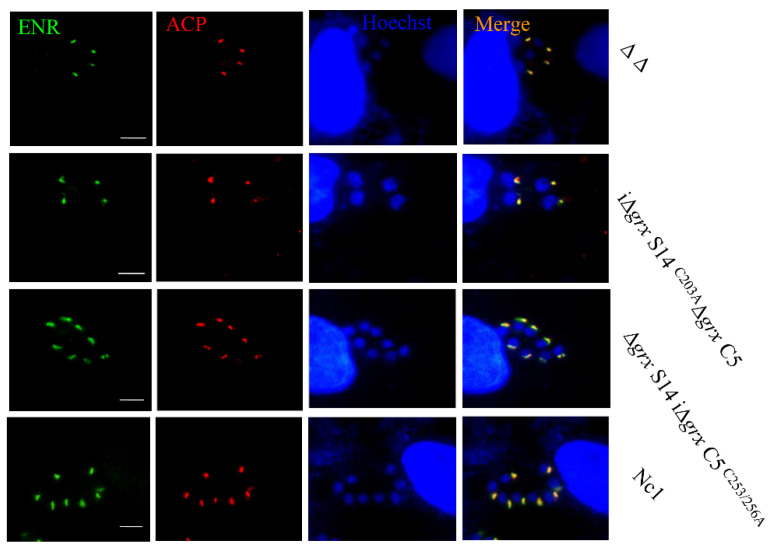
Location of apicoplast proteins in grx S14 and grx C5 deletion and mutant strains. Locations of ENR (green) and ACP (red) in comΔ*grx* S14^AXXS^Δ*grx C5,* Δ*grx* S14comΔ*grx* C5^AXXA^, comΔ*grx* S14Δ*grx* C5, Δ*grx* S14comΔ*grx* C5, Δ*grx* S14Δ*grx* C5 and Nc1 strains were detected by IFA. Parasite shapes were visualized with anti-NcSRS2 (red); nuclear DNA was stained with Hoechst (blue). Scale bar = 5 μm.

Previous research showed that thioredoxins contributed to the control of protein trafficking to the apicoplast [17]. The localization of the apicoplast proteins, the enoyl acyl carrier protein reductase (ENR) and acyl carrier protein (ACP), was observed in Nc1, Δ*grx* S14Δ*grx* C5, comΔ*grx* S14^AXXS^Δ*grx* C5, Δ*grx* S14comΔ*grx* C5^AXXA^, Δ*grx* S14comΔ*grx* C5 and comΔ*grx* S14Δ*grx* C5. As the localization of the apicoplast proteins ENR and ACP in the deletion or mutant strain was normal (Figure 7), GRX S14 and GRX C5 do not appear to influence protein trafficking to the apicoplast.

### 2.5. Double-Gene Depletion Affects the Expression of Several Proteins

**Figure 8:**
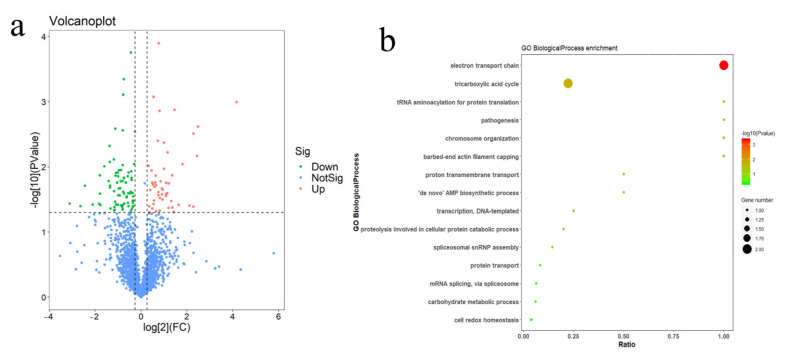

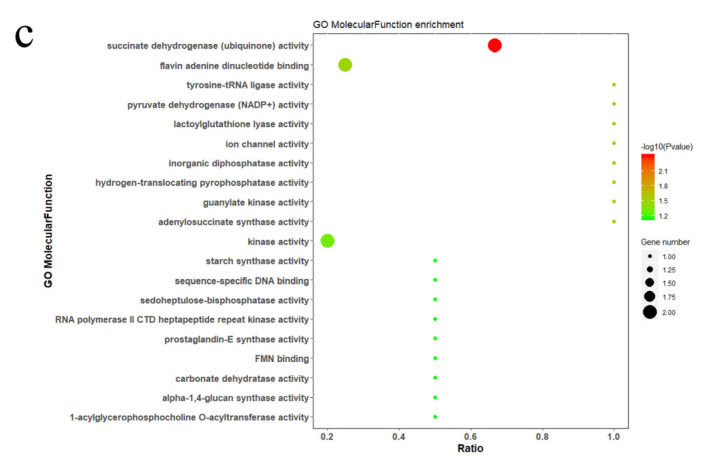
The comparative proteome of Δ*grx* S14Δ*grx* C5 and Nc1. (**a**) Volcano plots showing log_2_ protein ratios vs. −log_2_ *p* values of the global proteome in Δ*grx* S14Δ*grx* C5 compared to Nc1 parasites. (**b**) Gene Ontology (GO) analysis of proteins with downregulated expressions in Δ*grx* S14Δ*grx* C5 compared to Nc1 parasites based on biological process and (**c**) molecular function (see Supplementary Dataset S1).

To explore which pathways of the parasite are affected by double-gene depletion, we performed a comparative proteomic analysis between Δ*grx* S14Δ*grx* C5 and the Nc1 parasites. The Δ*grx* S14Δ*grx* C5 parasites showed a significant downregulation of 29 proteins (fold change > 2, *p*-value < 0.05, Figure 8a). According to GO enrichment analysis, the biological functions of the proteins with downregulated expressions were mainly related to the electron transport chain and tricarboxylic acid cycle (Figure 8b,c).

## 3. Discussion

GRXs are ubiquitous oxidoreductases that maintain a cellular redox equilibrium and catalyse thiol-disulphide exchange reactions by utilizing GSH [5]. GRXs are classified as monothiol (CXXS) or dithiol (CXXC) GRXs depending on the number of cysteine residues present in the redox active site [7,8]. The biological functions of GRXs include DNA/RNA synthesis, Fe–S cluster assembly, cell signal transduction, apoptosis and cell proliferation [5,6]. Only a few GRXs from parasites have been reported, mainly for trypanosomes and malaria parasites [10,15].

We identified five putative GRXs in *N. caninum*. Our previous study showed that the GRX1 (NcGRX1) and GRX3 (NcGRX3) of *N. caninum* are located in the cytoplasm [16] and NcGRX5 in mitochondria. In the present study, we found that GRX S14 and GRX C5 localize to the apicoplasts. The apicoplast is an essential, nonphotosynthetic plastid found in related apicomplexan pathogens [18,19]. This organelle is the product of a secondary endosymbiosis event and is homologous to the chloroplasts of algae and plants [19]. Therefore, the functions of GRX S14 and GRX C5 might be similar to those of plant GRXs. GRXs have a crucial role in the developmental process of *A. thaliana*. For example, the lack of Class I GRX C1 and GRX C2 proteins in *Arabidopsis* can lead to an impaired embryonic development and even cause death [20]. *Arabidopsis* GRX S14, GRX S15, GRX S16 and GRXS17 are Class II GRXs. GRX S14 is composed of two domains: an N-terminal domain with an endonuclease activity and a C-terminal domain with a GRX motif [21]. The silencing of tomato GRXS16 results in an increased sensitivity to osmotic pressure [22]. GRX S17 consists of a TRX-like domain and three GRX domains and plays a key role in controlling plant development. The *A. thaliana* GRX S17 mutant strain displays a slowed primary root growth and impaired flowering at 28 °C [23]. This mutant strain exhibits severe nutritional and reproductive development impairment under a long-day photoperiod [24]. GRX S14 and GRX S15 are associated with oxidative stress, high temperature and arsenic exposure [25].

One study showed that NcGRX1 is important for microneme protein-mediated parasite growth, but that NcGRX3 deficiency does not affect parasite growth [16]. Our study showed that the deletion of GRX S14 or GRX C5 alone did not affect the growth of *N. caninum*, which was consistent with previous research on *Arabidopsis* [25]. GRX C5 has a CPFC active site and is homologous to *Arabidopsis* Class I GRX C5. AtGRXC5, which has two forms, is expressed in *Escherichia coli*. The monomeric apoprotein of AtGRXC5 exhibits a deglutathionylation activity in mediating the recycling of the plastidial methionine sulfoxide reductase B1 and peroxiredoxin IIE. The dimeric holoprotein of AtGRXC5 incorporates a (2Fe–2S) cluster [26]. In our study, single-gene deletion did not affect the growth of parasites, whereas the simultaneous deletion of both apicoplast GRXs reduced their growth. These results revealed that GRX S14 or GRX C5 might have a synergistic effect during parasite growth. In addition, parasites with a mutated cysteine in the CPFC motif of GRX C5 or the CGYS motif of GRX S14 displayed a reduced growth, indicating that these motifs were the key active sites of each GRX.

*Arabidopsis* GRX S14 is a new signalling molecule in plants that regulates the Ca^2+^ transport activity of CAX1 by interacting with the N-terminal region of CAX1 (cation exchanger) [27] and protecting against protein oxidative damage [28]. *Arabidopsis* and poplar GRX S14 are located in the chloroplast and form a bridge with the (2Fe–2S) cluster and two external GSH ligands. GRX S14 is used as a scaffold protein for (2Fe–2S) cluster assembly because it transfers the complete cluster to the receptor protein regulated by GSH [29,30]. Our previous research revealed that *N. caninum* GRX1 deficiency decreases the ratio of reduced GSH/GSSG, causing a significant accumulation of hydroxyl radicals in parasites, and increases the number of apoptotic cells under oxidative stress (H_2_O_2_) conditions [16]. In the present study, GRX S14 and GRX C5 double-gene deletion did not affect the GSH/GSSG ratio of parasites, nor did it alter levels of ROS and OH. Thus, GRX S14 and GRX C5 may not have important roles in regulating the redox balance in *N. caninum*.

In addition, glutaredoxin and thioredoxin are oxidoreductases that together maintain a redox balance in cells. Previous studies have found that thioredoxin 1 of *T. gondii* is located in the apicoplast and involved in the control of protein trafficking to this organelle [17]. However, our results showed that a lack of both GRX proteins or mutations in the key active site did not affect localization of the apicoplast proteins ACP and ENR, indicating that the apicoplast GRX might not be involved in apicoplast protein import.

Apicoplast processes involve multiple metabolic pathways, including the synthesis of haem, type II fatty acids, and isoprenoid precursors, among others [31]. After the double deletion of *grx* S14 and *grx* C5, only 29 proteins were downregulated more twice, and no known apicoplast proteins were identified. Surprisingly, the downregulated proteins are involved in the mitochondrial electron transport chain (ETC) and TCA cycle. This result suggests that GRX S14 and GRX C5 may not be involved in apicoplast function but instead may be related to the ETC and TCA cycle. Regardless, the mechanism by which apicoplast proteins are involved in the mitochondrial ETC or TCA process remains unclear. In summary, we identified two new GRXs localized to the apicoplasts. Double-gene deletion resulted in a significant growth defect and caused the downregulation of the expression of proteins involved in the electron transport chain and TCA cycle.

## 4. Materials and Methods

### 4.1. Parasites and Cell Culture

The *N. caninum* wild-type (WT) strain (Nc1) was used as the parental parasite for genetically engineered strains. Parasites were grown in vitro on HFF cells using 2% FBS at 37 °C and 10% CO_2_.

### 4.2. Construction of Transgenic Parasite Lines

The CRISPR/Cas9 system was used to generate *grx* S14 and *grx* C5 deficiency (Δ*grx* S14 and Δ*grx* C5 parasites). The EuPaGDT Library in ToxoDB was used to design the gRNA targeting sites of the plasmids pCRISPR-CAS9-*grx* S14 and pCRISPR-CAS9-*grx* C5. The basic plasmid template was CRISPR-CAS9-GRX1, which was constructed in our previous study [16]. Cas9 upstream and downstream fragments containing gRNA sequences were amplified and ligated by seamless cloning (Vazyme Biotech, Co., Ltd., Nanjing, China). For disruption of the *grx* S14 locus, dihydrofolate reductase (DHFR) was inserted into the 3′ flank and 5′ flank of the *grx* S14 regions and ligated into the plasmid backbone carrying ampicillin resistance. For construction of homologous recombinant plasmids of *grx* S14 (p5′*grx* S14-DHFR-3′*grx* S14), the 3′ flanking and 5′ flanking sequences of the *grx* S14 gene were amplified from genomic DNA of Nc1 parasites. The pCRISPR-CAS9-*grx* S14 and p5′*grx* S14-DHFR-3′*grx* S14 plasmids were co-transfected into Nc1 parasites, and the parasites were screened using pyrimethamine. Monoclonal screening was carried out by a limited dilution method, with reference to a previous study [32]. Δ*grx* S14 parasites were identified by PCR followed by sequencing. Construction of Δ*grx* C5 parasites was the same as that for Δ*grx* S14. For generation of double-gene knockout parasites (Δ*grx* S14Δ*grx* C5), the p5′*grx* C5-CAT-3′*grx* C5 homologous plasmid was constructed in the same way. The pCRISPR-CAS9-*grx* C5 and p5′*grx* C5-CAT-3′*grx* C5 homologous plasmids were co-transfected into Δ*grx* S14 parasites and then screened with chloramphenicol drugs. Finally, primers were designed to identify the monoclonal strains.

For the generation of Δ*grx* S14Δ*grx* C5 complemented parasites (Δ*grx* S14comΔ*grx* C5 and comΔ*grx* S14Δ*grx* C5), the UPRT gene was disrupted by the UPRT-specific CRISPR-Cas9 plasmid and replaced with the p5′UPRT-Tubulin promoter-DHFR-*grx* S14/*grx* C5-HA-3′UPRT sequence, as described previously [16]. The *grx* S14 and *grx* C5 expression sequences were amplified using Nc1 cDNA and inserted into the p5′UPRT-Tubulin promoter-DHFR-Grx1-HA-3′UPRT plasmid (preserved in the Key Laboratory of Animal Parasitology, Beijing City, China) by seamless cloning (Vazyme Biotech, Co., Ltd., Nanjing, China). The p5′UPRT-Tubulin promoter-DHFR-*grx* S14/*grx* C5-HA-3′UPRT and UPRT-specific CRISPR-Cas9 plasmids were co-transfected into the Δ*grx* S14Δ*grx* C5 parasites and then screened with fluorodeoxyribose (FUDR) drugs. The construction strategy for the overexpression strain was the same as that for the complementary strain. The homologous recombinant plasmid and CRISPR/CAS9-UPRT plasmids were co-transfected into Nc1 parasites. For construction of cysteine mutant parasites, the cysteine of *grx* S14 in the CGYS motif (comΔ*grx* S14^AXXS^Δ*grx* C5) and *grx* C5 in the CPFC motif (Δ*grx* S14comΔ*grx* C5^AXXA^) were mutated to alanine. The construction method was the same as that for the complemented parasites. The mutant sequences of *grx* C5^AXXA^ and *grx* S14^AXXS^ were checked by PCR.

To obtain GRX1-HA parasites, we constructed a pLIC-DHFR-*grx* S14-HA plasmid for inserting a 3×HA tag into the *grx* S14 gene 3′ end. The 3′ flank and 5′ flank regions of *grx* S14 were amplified from the DNA of the Nc1 parasites and inserted into the backbone of the pLIC-DHFR-HA plasmid using seamless cloning (Vazyme Biotech, Co., Ltd., Nanjing, China). The construction strategy of pCRISPR-CAS9 was consistent with the above method. The pLIC-DHFR-*grx* S14-HA plasmid and pCRISPR-CAS9 plasmids were co-transfected into Nc1 parasites and screened with pyrimethamine.

### 4.3. Immunoblotting and Immunofluorescence Assays

Immunoblotting was performed as previously reported [16]. Briefly, tachyzoites were collected and lysed with RIPA lysis buffer (Huaxinbio, Beijing, China). Mouse anti-HA (MAb, 1:5000, Sigma, St. Louis, MO, USA) and anti-actin (1:5000) were used as primary antibodies. For IFA, tachyzoite-infected HFFs were treated with 4% paraformaldehyde (PFA) and then permeated with 0.25% Triton X-100, followed by blocking with 3% BSA. Subsequently, the samples were incubated with primary mouse anti-HA (1:100), mouse anti-ACP (1:300), rabbit anti-ENR (1:200) and rabbit anti-SRS2 (1:400) for 1 h; secondary FITC- or Cy3-conjugated antibodies were used for labelling. DNA was stained with Hoechst 33258 (Sigma, St. Louis, MO, USA). Images were observed using a Leica confocal microscope system (Leica, TCS SP52, Wetzlar, Hesse, Germany).

### 4.4. Phenotypic Assays

#### 4.4.1. Plaque Assays

HFFs were grown in 12-well plates for three days, after which 300 tachyzoites were inoculated into the cells. After the culture was left undisturbed for 9 days, the infected HFFs were fixed with 4% PFA and observed by crystal violet staining. The plaque area was counted based on pixels using Photoshop C6S software (Adobe, San Jose, CA, USA), and data were compiled from three independent experiments.

#### 4.4.2. Invasion Assay and Intracellular Replication Assay

Parasites were used to infect fresh HFF cells seeded on coverslips for 1 h and washed with PBS to remove noninvaded parasites. After 24 h, invasion and intracellular replication were evaluated by IFA using rabbit anti-SRS2 antibodies and Hoechst staining. For the invasion assay, the percentage of invasion was represented as the number of vacuoles per host cell. For the intracellular replication assay, the number of parasites per vacuole for each strain was determined by counting at least 100 vacuoles under a fluorescence microscope (Olympus Co., Tokyo, Japan).

#### 4.4.3. Egress Assay

HFF cells seeded on coverslips were infected with parasites for 36 h. Egress was triggered with 2 μM A23187 Ca^2+^ ionophore (Sigma, St. Louis, MO, USA) for 3 min and then fixed with PFA. The ruptured vacuoles and unbroken vacuoles were counted per slide to evaluate the egress rate. One hundred random fields of vision were counted per slide to evaluate the egress ratio. Three independent experiments were performed.

#### 4.4.4. *N. caninum* Mouse Infection

BALB/c mice purchased from Merial Animal Health Co., Ltd. (Beijing, China), raised in a barrier environment in sterile cages and fed sterilized food and clean water ad libitum. Animals were acclimated to these conditions for one week prior to the experiment. BALB/c mice (five mice per strain) were infected intraperitoneally with 8 × 10^6^ parasites. The period for observing the survival was 30 days. The mice were humanely euthanized after 30 days.

#### 4.4.5. Detection of Reactive Oxygen Species

The fluorescence intensity of parasites with green fluorescence generated by DCF can reflect the ROS level in parasites. The ΔΔ*grx* S16 *grx* C5 and WT parasites were grown in HFF cells seeded on coverslips. After 24 h, the parasites were purified and treated with 30 μM DCFH-DA at 37 °C for 1 h and resuspended in 100 μL of PBS after two washes. Finally, 100 μL of parasites was analysed by flow cytometry. The mean fluorescence intensity was used to indicate the amount of ROS in the parasites.

#### 4.4.6. Detection of Hydroxyl Radicals

Parasites were grown in HFF cells for 24 h and filtered using 5-μm polycarbonate membranes. The concentration of hydroxyl radicals in parasites was detected using a hydroxyl radical detection kit using hydroxyphenyl fluorescein (2-[6-(4′-hydroxy) phenoxy-3H-xanthen-3-on-9-yl] benzoic acid, HPF) according to the manufacturer’s instructions (Genmed Scientific, Inc., Boston, MA, USA) and a microplate reader (Synergy H(1), Biotek, Winooski, VT, USA) at 590 nm.

#### 4.4.7. GSH and GSSG Determination

A total of 1×10^7^ parasites of each strain were harvested and lysed by freezing in liquid nitrogen and thawing at 37 °C in three cycles. The supernatant of each sample was collected for GSH and GSSG measurement using a GSH and GSSG Assay Kit according to the manufacturer’s instructions (Beyotime, Shanghai, China).

#### 4.4.8. Comparative Proteomics Analysis

Intracellular tachyzoites of the Nc1 and ΔΔ*grx S14grx C5* strains cultured in HFF cells were subjected to sequential syringe lysis and filtered through a 5-μm membrane. The samples were washed twice with ice-cold PBS, minced individually with liquid nitrogen and sent to Shanghai Applied Protein Technology Co., Ltd. for global proteome analysis (Shanghai, China).

### 4.5. Statistical Analysis

Graphs were created using GraphPad Prism (San Diego, CA, USA). All bar graphs and scatter plots depict the mean with standard deviations shown as error bars. All data were analysed with the *t*-test and two-way ANOVA. *p* values are represented by asterisks in the figures as follows: * *p* < 0.05, ** *p* < 0.01, and *** *p* < 0.001. We consider all *p*-values < 0.05 to be significant.

## Data Availability

All datasets generated for this study are included in the manuscript/Supplementary files.

## Supplementary Materials

The following are available online at https://www.mdpi.com/article/10.3390/ijms222111946/s1.
