# Supplementary figures and images for: Identification and Function of Apicoplast Glutaredoxins in Neospora caninum
Source: Int J Mol Sci. 2021 Nov 4;22(21):11946. doi: 10.3390/ijms222111946 (PMC8584781; doi:10.3390/ijms222111946)

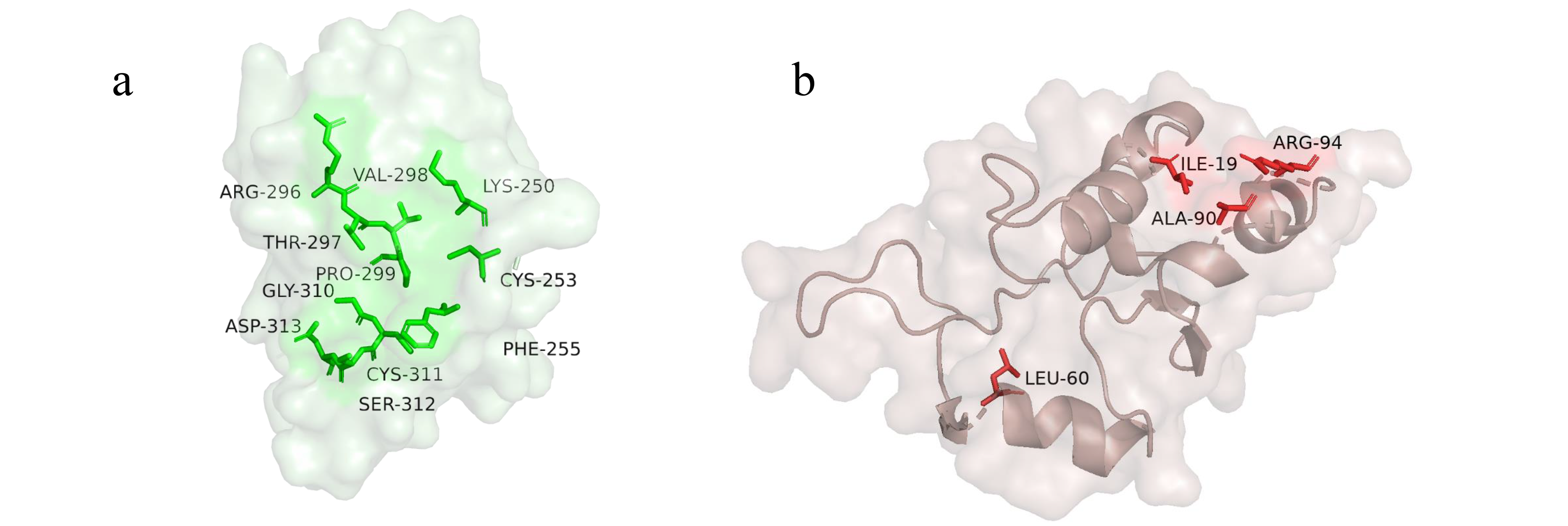

Supplement: Supplementary file 1 [file ijms-22-11946-s001.zip › Figure S1.tif]

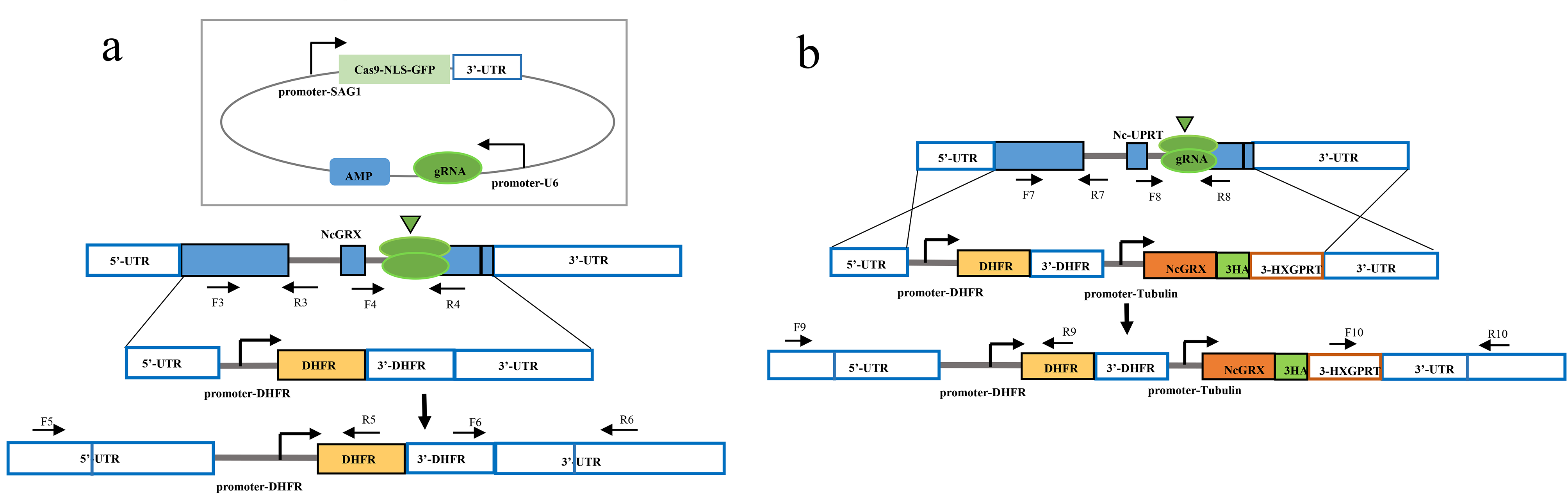

Supplement: Supplementary file 1 [file ijms-22-11946-s001.zip › Figure S2.tif]
